# Gesture imitation performance in community‐dwelling older people: assessment of a gesture imitation task in the screening and diagnosis of mild cognitive impairment and dementia

**DOI:** 10.1111/psyg.13086

**Published:** 2024-01-30

**Authors:** Akihiro Takasaki, Mamoru Hashimoto, Ryuji Fukuhara, Shizuka Sakuta, Asuka Koyama, Tomohisa Ishikawa, Shuken Boku, Manabu Ikeda, Minoru Takebayashi

**Affiliations:** ^1^ Graduate School of Medical Sciences Kumamoto University Kumamoto Japan; ^2^ Department of Neuropsychiatry Kindai University Faculty of Medicine Osakasayama Japan; ^3^ Department of Psychiatry Kagoshima University Graduate School of Medical and Dental Sciences Kagoshima Japan; ^4^ Faculty of Social Welfare Kumamoto Gakuen University Kumamoto Japan; ^5^ Department of Psychiatry Arao Kokoronosato Hospital Arao Japan; ^6^ Department of Neuropsychiatry, Faculty of Life Sciences Kumamoto University Kumamoto Japan; ^7^ Department of Psychiatry Osaka University Graduate School of Medicine Suita Japan

**Keywords:** Alzheimer's disease, cube‐copying, dementia with Lewy bodies, gesture imitation, screening test, visuospatial/visuoconstructive function

## Abstract

**Background:**

Gesture imitation, a simple tool for assessing visuospatial/visuoconstructive functions, is reportedly useful for screening and diagnosing dementia. However, gesture imitation performance in healthy older adults is largely unknown, as are the factors associated with lower performance. To address these unknowns, we examined the gesture imitation performance of a large number of community‐dwelling older adults aged ≥65 years in Arao City, Kumamoto Prefecture (southern Japan).

**Methods:**

The examiner presented the participants with eight gesture patterns and considered it a success if they could imitate them within 10 s. The success rate of each gesture imitation was calculated for three diagnostic groups: cognitively normal (CN) (*n* = 1184), mild cognitive impairment (MCI) (*n* = 237), and dementia (*n* = 47). Next, we reorganised the original gesture imitation battery by combining six selected gestures with the following scoring method: if the participants successfully imitated the gestures, immediately or within 5 s, two points were assigned. If they succeeded within 5–10 s, one point was assigned. The sensitivity and specificity of the battery were investigated to detect the dementia and MCI groups. Factors associated with gesture imitation battery scores were examined.

**Results:**

Except one complex gesture, the success rate of imitation in the CN group was high, approximately 90%. The sensitivity and specificity of the gesture imitation battery for discriminating between the dementia and CN groups and between the MCI and CN groups were 70%/88%, and 45%/75%, respectively. Ageing, male sex, and a diagnosis of dementia or MCI were associated with lower scores on the gesture imitation battery.

**Conclusion:**

Gesture imitation tasks alone may not be sufficient to detect MCI. However, by combining gestures with set time limits, gesture imitation tasks can be a low‐burden and effective method for detecting dementia, even in community medicine, such as during health check‐ups.

## INTRODUCTION

Social interest in early intervention and prevention of dementia has increased in recent years due to the growing number of people with dementia. Several disease‐modifying drugs for Alzheimer's disease (AD) have also been introduced. Development of assessment tools that enable simple screening for early dementia, possibly at the mild cognitive impairment (MCI) stage, is needed not only in clinical medicine but also in community medicine. Visuospatial cognitive dysfunction is one of the most common cognitive deficits in patients with dementia, along with deficits in complex attention, executive function, learning and memory, language, and social cognition (Diagnostic and Statistical Manual of Mental Disorders, 5th edition (DSM‐V)). Visuospatial cognitive impairments appear early in dementia due to AD (ADD) and dementia with Lewy bodies (DLB),^1^, ^2^ while in frontotemporal dementia, visuospatial cognitive function is relatively preserved throughout the course of the disease.^3^ Therefore, appropriate assessment of visuospatial cognitive function can lead to an early and differential diagnosis of dementia.

The figure‐copying task is a classic measure of visuospatial/visuoconstructive function,^4^, ^5^ which has been incorporated into dementia screening tests such as the Mini‐Mental State Examination (MMSE).^6^, ^7^ Shimada *et al*.^8^ reported that the Necker cube‐copying task may be useful in identifying patients with mild ADD. Recently, several studies have reported the usefulness of gesture imitation tasks for assessing visuospatial/visuoconstructive function in the screening and differential diagnosis of dementia.^9^, ^10^, ^11^, ^12^, ^13^, ^14^, ^15^, ^16^, ^17^ Gesture imitation is a simple task which requires the examinee to imitate finger configurations and hand postures made by the examiner. Compared with the figure‐copying tasks, gesture imitation tasks have the advantage of being performed easily at the bedside, without the requirement for writing tools. Due to its simplicity, the gesture imitation task is expected to be one of the most adoptable screening methods for dementia and MCI; however, several issues need to be addressed before its standard application.

First, insufficient data are available on gesture imitation tasks in healthy older people. Few studies have reported gesture imitation performance in healthy subjects, but they included people under the age of 65.^18^, ^19^ Thus, when older adults fail to imitate gestures, it cannot be judged whether the decline is pathological or age‐related. Second, there are no standardised methods for categorising gesture types and scoring, as previous studies have used various approaches. This variability in methodologies impedes the ability to determine which methods are more effective for diagnosis and screening in MCI and dementia. Third, none of the previous studies, reporting the usefulness of gesture imitation tasks in dementia screening and diagnosis, have clarified whether they are also effective for MCI. Fourth, although poor education and female sex have been reported to be associated with poor figure‐copying performance,^8^, ^20^, ^21^ little is known about the effects of these factors on gesture imitation performance.

In this study, we performed two types of gesture imitation methods, incorporating several gestures previously reported as useful in dementia diagnosis and screening, in a large population of community‐dwelling people aged ≥65 years. Our aim was to address the following questions. (1) How accurately can healthy older adults imitate gestures? (2) What types of gestures and scoring methods are the most useful in the screening and diagnosis of dementia and MCI? (3) What factors contribute to poor gesture imitation performance?

## METHODS

### Study design and participants

A total of 1577 community‐dwelling older adults in Arao City, Kumamoto Prefecture (southern Japan), were enrolled between November 2016 and March 2017. This cross‐sectional analysis using baseline data was part of the Japan Prospective Studies Collaboration for Ageing and Dementia (JPSC‐AD), which is designed to enrol approximately 10 000 community‐dwelling adults aged ≥65 years, from eight sites in Japan, to explore the genetic and environmental risk factors for dementia.^22^ Participants were excluded from this analysis if they diagnosed depression according to the DSM‐IV, severe neurological impairments that interfered with the performance of neuropsychological tests, no brain magnetic resonance imaging (MRI) data, or missing data.

Neuropsychiatrists diagnosed MCI or dementia in participants, according to the JPSC‐AD diagnostic procedure^22^ based on the results of questionnaires, neurological and neuropsychological assessments, and brain MRI.^23^, ^24^ MCI was diagnosed according to Petersen's criteria,^25^ dementia according to the DSM‐III‐R (Revised Edition), and dementia subtypes according to standard criteria.^22^ The National Institute of Neurological and Communicative Disorders and Stroke and Alzheimer's Disease and Related Disorders Association criteria (NINCDS‐ADRDA)^26^ were used for diagnosing ADD, National Institute of Neurological Disorders and Stroke‐Association International pour la Recherche et l'Enseignement en Neurosciences criteria (NINDS‐AIREN)^27^ for vascular dementia, and Third Consensus Report of the Dementia with Lewy Bodies (DLB) Consortium^28^ for DLB. Participants without MCI or dementia were considered cognitively normal (CN).

The Research Ethics Committee of Kumamoto University (Kumamoto, Japan; approval number: GENOME‐333) approved this study. All participants provided written informed consent before data collection, in accordance with the Declaration of Helsinki.

### Gesture imitation tasks

**Figure 1:**
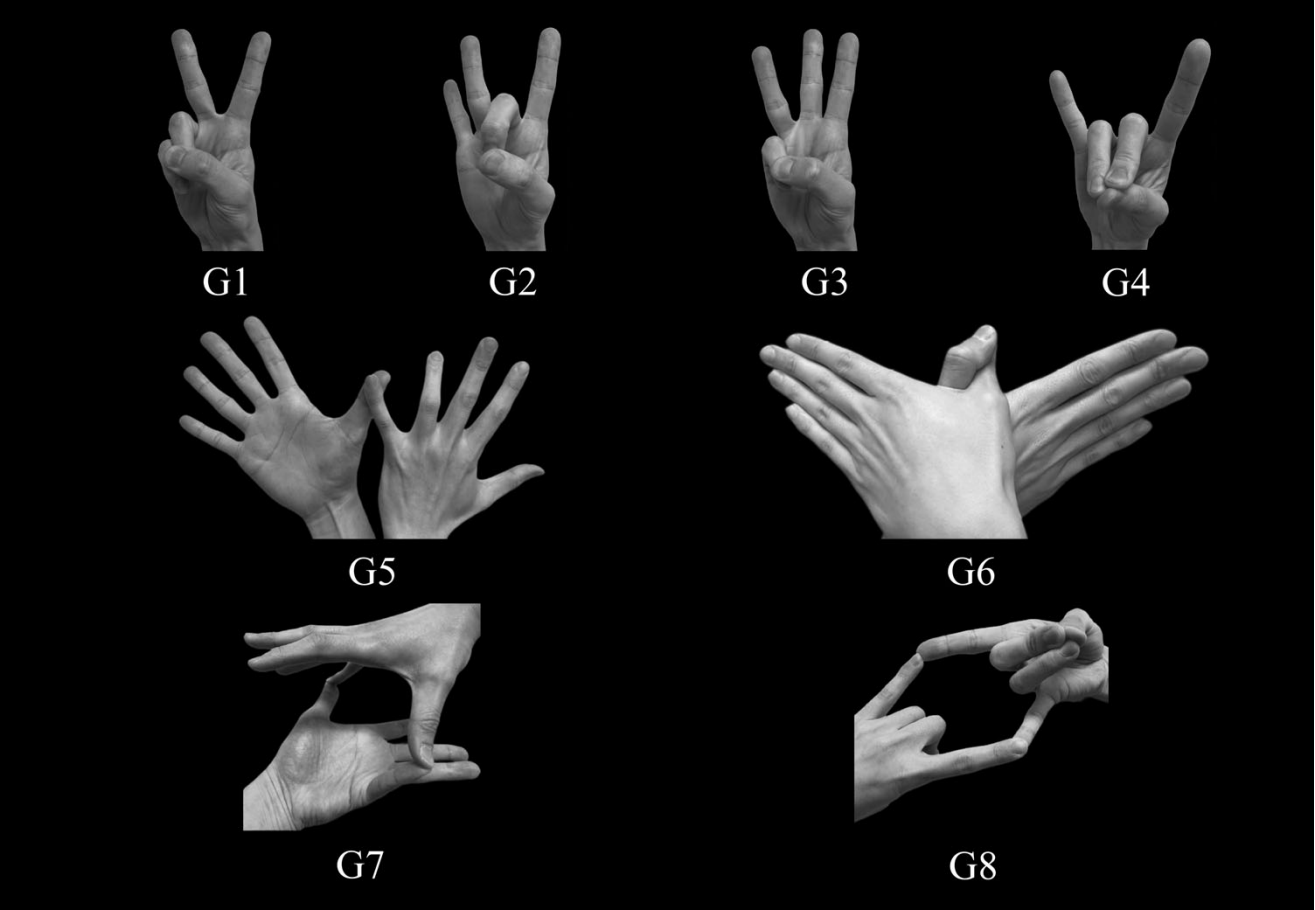
Examiner's demonstration of the unimanual and bimanual gestures. G1–G4 are unimanual and G5–G8 are bimanual gestures. In Japan, G1, G3, G4, and G6 are considered meaningful gestures, whereas G2, G5, G7, and G8 are not. G1: scissors or peace sign; G3: number 3; G4: a fox; G6: a butterfly or pigeon.

Eight gesture patterns reported in previous studies^10^, ^11^, ^12^, ^13^ were used to assess gesture imitation performance (Fig. 1). Gestures G1–G4 are unimanual, and G5–G8 are bimanual. In Japan, G1, G3, G4, and G6 are considered meaningful gestures, whereas G2, G5, G7, and G8 are not. G1 refers to scissors or the peace sign. The Japanese use G1 to express joy, happiness, and affection, rather than victory. G3, G4, and G5 refer to the number three, fox, and butterfly or pigeon, respectively.

Each participant sat in front of the examiner, a trained clinical psychologist, and was given the instructions, ‘Watch carefully. After I make the gesture, please imitate it.’ G1–G4 were performed by the examiner with the right hand, and the participant was allowed to perform the gestures with either hand. After unimanual gesture imitation, participants were asked to imitate bimanual gestures with both hands. The examiner only showed completed bimanual gestures and did not show the process of correctly performing the gesture with both hands. The examiner demonstrated these gestures in numerical order (G1–G8) and presented each gesture for 10 s, without further instructions. If the participant successfully imitated the gesture within 10 s, the examiner stopped the demonstration and moved on to the next gesture.

The gesture imitation tasks were scored using two methods. In Method A, if the participant successfully imitated a gesture immediately or within 5 s, two points were assigned. If the participant succeeded within 5–10 s, one point was assigned. In Method B, if the participant succeeded within 10 s, one point was assigned. If the participant imitated the unimanual gestures (G1–G4) with the back of the hand facing the examiner, we considered it an error pattern and assigned no points. We also assigned no points when the participant imitated G6 with the palms of both hands facing the examiner.

### Clinical evaluation

**Figure 2:**
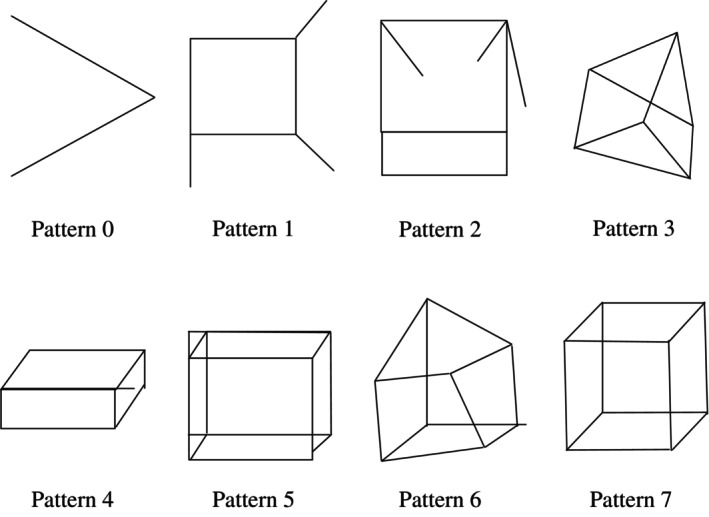
Criteria for the Necker cube‐copying task by Shimada *et al*.^8^ Pattern 7 is a perfect copy of the Necker cube, while patterns 0–6 are incomplete. The smaller the number is, the more imperfect it is. Each pattern was prepared by the author (AT) with reference to the study by Shimada *et al*.^8^

Trained clinical psychologists administered neuropsychological tests to all participants. Global cognitive function was measured using the MMSE. The Necker cube‐copying task was administered to assess visuospatial/visuoconstructive function in comparison to the gesture imitation tasks. The results of the Necker cube‐copying task were scored by two authors (AT and AK) according to the criteria described by Shimada *et al*.^8^ (Fig. 2). The Necker cube‐copying task scores ranged from zero to seven. Only a successful copy was assigned seven points. The Geriatric Depression Scale 15 item version (GDS)^29^ was administered to assess each participant's subjective mood.

### Statistical analysis

Categorical demographic variables and the success rate of each gesture imitation and Necker cube‐copying were compared among the three diagnostic groups using the χ^2^ test and Fisher's exact test. For comparisons between two groups, the χ^2^ test and Fisher's exact test were performed for the CN and MCI groups, CN and dementia groups, and MCI and dementia groups. Continuous demographic variables and neuropsychological test scores were compared among the three diagnostic groups using one‐way analysis of variance and Bonferroni *post hoc* tests.

We calculated the area under the curve (AUC) in the receiver operating characteristic curves as a measure of the predictive value of each gesture imitation, scored using Methods A or B, for detecting dementia and MCI groups. We then selected the scoring method that showed the highest predictive value and excluded gestures that were not significant predictive indicators of dementia or MCI. Next, we calculated the AUC for the reorganised gesture imitation battery, which combined the selected gestures and scoring method, to detect dementia or MCI.

To examine factors associated with gesture imitation performance, such as age, sex, and years of education, we conducted multiple regression analysis using gesture imitation battery scores as the dependent variable. For comparison, the same analysis was conducted on the Necker cube‐copying task scores.

All tests were two‐tailed, and the significance level was set at *P* < 0.05. Bonferroni corrections were used to correct for multiple comparisons; for example, the significance level for comparisons among the three diagnostic groups was *P* < 0.017 (0.05/3). All statistical analyses were performed using SPSS 25.0 J for Windows (IBM SPSS Japan, Tokyo, Japan).

## RESULTS

**Figure 3:**
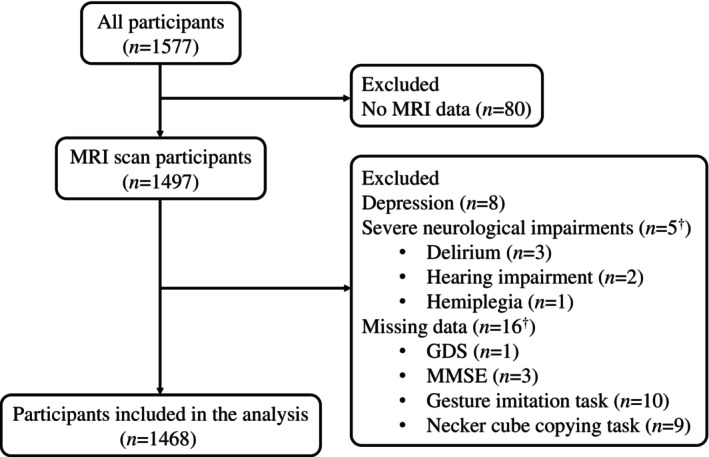
Study enrolment flowchart. MRI, magnetic resonance imaging; GDS, Geriatric Depression Scale; MMSE, Mini‐Mental State Examination. ^†^Including overlaps, not the sum of the following items.

A total of 109 individuals were excluded (Fig. 3). Thus, 1468 participants (892 women; mean age ± standard deviation: 74.2 ± 6.4) were included in the analysis. Table 1 shows the demographic characteristics of the participants. Of these, 1184 were CN, 237 had MCI, and 47 had dementia (41 had ADD, four had vascular dementia, one had DLB, and one had unclassified dementia). There were significant differences in age (*F* = 97.9, *P* < 0.001), sex (χ^2^ = 12.9, *P* = 0.001), years of education (χ^2^ = 57.3, *P* < 0.001), GDS total score (*F* = 27.7, *P* < 0.001), and MMSE total score (*F* = 642.9, *P* < 0.001) among the three diagnostic groups.

**Table 1:** Demographic characteristics of participants

	CN, *n* = 1184	MCI, *n* = 237	Dementia, *n* = 47	*F*/χ^2^	*P*‐value
Age (years)	73.1 ± 6.0	77.9 ± 6.3	81.5 ± 6.8	97.9^†^	*P <* 0.001*, §, ¶, ††
Sex (female), %	63.0	51.5	51.1	12.9^‡^	*P* = 0.001^*^, ^§^
Years of education (≥ 10), %	76.2	57.8	40.4	57.3^‡^	*P* < 0.001^*^, ^§^, ^¶^, ^††^
GDS (/15)	2.1 ± 2.1	3.1 ± 2.8	3.8 ± 2.6	27.7^†^	*P* < 0.001^*^, ^§^, ^¶^
MMSE (/30)	27.9 ± 2.0	24.5 ± 2.5	18.3 ± 3.4	642.9^†^	*P* < 0.001^*^, ^§^, ^¶^, ^††^

* *P* < 0.017 (0.05/3) after Bonferroni correction.

^†^ *F* = one‐way analysis of variance; mean ± standard deviation.

^‡^ χ^2^ = The χ^2^ test.

^§^ Significant difference between CN and MCI.

^¶^ Significant difference between CN and dementia.

^††^ Significant difference between MCI and dementia.

Abbreviations: CN, cognitively normal; MCI, mild cognitive impairment; GDS, Geriatric Depression Scale; MMSE, Mini‐Mental State Examination.

Table 2 shows the success rates for each gesture imitation and Necker cube‐copying among the three diagnostic groups. There were significant differences in the success rates of G3–G8 imitations and Necker cube‐copying among the three groups. G8 imitation had the lowest success rate, 63.4% even in the CN group, and only 25.5% in the dementia group. Success rate of each gesture imitation and Necker cube‐copying in the CN group of each age, sex, and educational level is given in Appendices S1–S3.

**Table 2:** Success rate of each gesture imitation and Necker cube‐copying task

	CN, *n* = 1184	MCI, *n* = 237	Dementia, *n* = 47	χ^2^	*P*‐value
Gesture imitation					
G1	99.9%	99.6%	100.0%	―^‡^	0.350
G2	97.6%	97.0%	95.7%	―^‡^	0.471
G3	99.3%	99.2%	93.6%	―^‡^	0.010^*^, ^¶^, ^††^
G4	98.3%	97.5%	80.9%	―^‡^	*P* < 0.001^*^, ^¶^, ^††^
G5	91.6%	85.7%	72.3%	―^‡^	*P* < 0.001^*^, ^§^, ^¶^, ^††^
G6	87.7%	76.4%	59.6%	44.1^†^	*P* < 0.001^*^, ^§^, ^¶^, ^††^
G7	89.9%	82.7%	53.2%	61.8^†^	*P* < 0.001^*^, ^§^, ^¶^, ^††^
G8	63.4%	49.8%	25.5%	39.4^†^	*P* < 0.001^*^, ^§^, ^¶^, ^††^
Necker cube‐copying	78.5%	58.2%	38.3%	74.5^†^	*P* < 0.001^*^, ^§^, ^¶^, ^††^

* *P* < 0.017 (0.05/3), after Bonferroni correction.

^†^ χ^2^ = The χ^2^‐test.

^‡^ Fisher's exact test.

^§^ Significant difference between CN and MCI.

^¶^ Significant difference between CN and dementia.

^††^ Significant difference between MCI and dementia.

Abbreviations: CN, cognitively normal; MCI, mild cognitive impairment.

Table 3 shows the AUC for each gesture imitation, scored using Method A or B, for detecting dementia or MCI. The AUCs for all gestures detecting dementia or MCI were higher with Method A than with Method B. Therefore, Method A was adopted for scoring with the gesture imitation battery. The predictive accuracy of G1 and G3 scored by either method, A or B, was not significant for detecting dementia or MCI. We, therefore, included G2 and G4–G8 in the gesture imitation battery. The maximum score on the battery was 12.

**Table 3:** Area under the curve of each gesture imitation scored by Method A or B for predicting dementia or MCI

	For the dementia group					For the MCI group				
Gestures	AUC	SE	*P*‐value	95% CI		AUC	SE	*P*‐value	95% CI	
				inf	sup				inf	sup
A‐method										
G1	0.518	0.044	0.670	0.432	0.605	0.503	0.021	0.869	0.463	0.544
G2	0.660	0.046	*P* < 0.001	0.570	0.751	0.520	0.021	0.331	0.479	0.561
G3	0.563	0.047	0.143	0.472	0.654	0.511	0.021	0.582	0.471	0.552
G4	0.678	0.047	*P* < 0.001	0.585	0.772	0.527	0.021	0.185	0.486	0.569
G5	0.693	0.042	*P* < 0.001	0.610	0.776	0.550	0.021	0.015	0.509	0.592
G6	0.719	0.041	*P* < 0.001	0.639	0.799	0.557	0.021	0.005	0.515	0.599
G7	0.702	0.045	*P* < 0.001	0.614	0.790	0.548	0.021	0.021	0.506	0.589
G8	0.709	0.034	*P* < 0.001	0.643	0.776	0.583	0.020	*P* < 0.001	0.544	0.622
B‐method										
G1	0.500	0.043	0.992	0.415	0.584	0.502	0.021	0.935	0.461	0.542
G2	0.509	0.044	0.833	0.424	0.595	0.503	0.021	0.902	0.462	0.543
G3	0.529	0.045	0.506	0.440	0.617	0.501	0.021	0.967	0.461	0.541
G4	0.587	0.048	0.042	0.494	0.680	0.504	0.021	0.838	0.464	0.545
G5	0.596	0.047	0.025	0.504	0.688	0.530	0.021	0.151	0.488	0.571
G6	0.640	0.046	0.001	0.550	0.731	0.556	0.021	0.006	0.515	0.598
G7	0.683	0.046	*P* < 0.001	0.593	0.774	0.536	0.021	0.081	0.494	0.577
G8	0.689	0.038	*P* < 0.001	0.615	0.764	0.568	0.021	*P* < 0.001	0.528	0.609

Abbreviations: MCI, mild cognitive impairment; AUC, area under the curve; SE, standard error; CI, confidence interval; inf, infimum; sup, supremum.

Method A: if the participants successfully imitated the gesture immediately or within 5 s, two points were assigned. If the participant succeeded within 5–10 s, one point was given. Method B: if the participant succeeded within 10 s, one point was assigned.

The AUC of the gesture imitation battery was 0.826 (*P* < 0.001) for the dementia group and 0.614 (*P* < 0.001) for the MCI group. Both AUCs were higher than those for a single gesture. Table 4 shows the sensitivity and specificity of the gesture imitation battery for detecting dementia or MCI according to different cut‐off points. The best cut‐off point for discriminating between the dementia and CN groups was 7/8, sensitivity was 70%, specificity was 88%, positive predictive value was 18%, and negative predictive value was 99%. The best cut‐off point for discriminating between the MCI and CN groups was 8/9, sensitivity was 45%, specificity was 75%, positive predictive value was 26%, and negative predictive value was 87%.

**Table 4:** Sensitivity and specificity of the gesture imitation battery with positive and negative predictive values for dementia or MCI

For the dementia group					For the MCI group				
Cut‐off	Se	Sp	PPV	NPV	Cut‐off	Se	Sp	PPV	NPV
0/1	0.02	1.00	1.00	0.96	0/1	0.00	1.00	0.00	0.83
1/2	0.02	1.00	1.00	0.96	1/2	0.00	1.00	0.00	0.83
2/3	0.06	1.00	0.38	0.96	2/3	0.00	1.00	0.00	0.83
3/4	0.19	0.99	0.47	0.97	3/4	0.02	0.99	0.33	0.83
4/5	0.32	0.98	0.43	0.97	4/5	0.05	0.98	0.39	0.84
5/6	0.45	0.96	0.31	0.98	5/6	0.08	0.96	0.30	0.84
6/7	0.62	0.93	0.26	0.98	6/7	0.17	0.93	0.33	0.85
7/8	0.70	0.88	0.18	0.99	7/8	0.27	0.88	0.30	0.86
8/9	0.77	0.75	0.11	0.99	8/9	0.45	0.75	0.26	0.87
9/10	0.81	0.60	0.07	0.99	9/10	0.57	0.60	0.22	0.87
10/11	0.89	0.39	0.05	0.99	10/11	0.73	0.39	0.19	0.88
11/12	0.96	0.23	0.05	0.99	11/12	0.86	0.23	0.18	0.89

Abbreviations: MCI, mild cognitive impairment; Se, sensitivity; Sp, specificity; PPV, positive predictive value; NPV, negative predictive value.

Table 5 shows the results of the multiple regression analysis for the scores on the gesture imitation battery and the Necker cube‐copying task. Age (*β* = −0.171, *P* < 0.001), sex (*β* = 0.059, *P* = 0.020), and diagnosis (*β* = −0.231, *P* < 0.001) were significantly associated with the gesture imitation battery scores. The Necker cube‐copying task scores were significantly associated with age (*β* = −0.135, *P* < 0.001), years of education (*β* = 0.147, *P* < 0.001), and diagnosis (*β* = −0.182, *P* < 0.001). We checked the variance inflation factor and found no collinearity between the variables.

**Table 5:** Multiple regression analyses of the gesture imitation battery and the Necker cube‐copying task scores

Independent variables	Gesture imitation battery		Necker cube‐copying task	
	*β*_STD_	*P‐*value	*β*_STD_	*P‐*value
Age	−0.171	*P* < 0.001	−0.135	*P* < 0.001
Sex^†^	0.059	0.020	−0.032	0.202
Years of education^‡^	−0.015	0.552	0.147	*P* < 0.001
Diagnosis^§^	−0.231	*P* < 0.001	−0.182	*P* < 0.001
*R*	0.333		0.337	
*R* ^2^	0.111		0.114	
Adjusted *R* ^2^	0.109		0.111	
*F*‐value	45.626	*P* < 0.001	46.828	*P* < 0.001

^†^ Reference = female.

^‡^ Reference = 10 years or more.

^§^ Cognitively normal = 0; mild cognitive impairment = 1; dementia = 2. *β*_STD_, standardised coefficient *β*.

## DISCUSSION

To the best of our knowledge, this is the first study to comprehensively examine gesture imitation performance in a large number of community‐dwelling older adults. We administered gesture imitation tasks to more than 1400 older adults, including more than 1100 CN older adults with no brain lesions and more than 200 MCI participants. The large dataset used in this study provides important insights into the usefulness as well as limitations of gesture imitation tasks in the screening and diagnosis of dementia and MCI.

The reorganised gesture imitation battery, in which we combined gestures and a scoring method to better predict participants with dementia and MCI, showed relatively high sensitivity (70%) and specificity (88%) but low positive predictive value (18%) for discriminating between participants with dementia and CN. While the specificity for participants with dementia was as high as previously reported,^10^, ^11^, ^12^, ^13^ the sensitivity was comparable to reports examining the effectiveness of gesture imitation for patients with mild or moderate dementia, but lower than that for patients with severe dementia.^10^, ^11^, ^12^ On the other hand, the extremely low positive predictive value seems to have been greatly influenced by the low prevalence of dementia in our study. While most previous studies were conducted on patients with dementia who visited medical institutions, the present study was conducted among community residents. In screening tests, it is important to minimise false‐negative results and identify false‐positive results before further investigation. Therefore, with its high specificity and negative predictive value for dementia, the gesture imitation battery could be a low‐burden and effective screening task for dementia, even in community medicine, such as health check‐ups.

The gesture imitation battery showed low sensitivity (45%) in discriminating between MCI and CN participants, indicating that more than half of MCI patients could be missed if this battery was used alone. Considering that the gesture imitation battery was specifically designed to measure visuospatial cognitive function and that more than half of patients with MCI are reported to be of the amnestic type, whose main symptom is memory impairment, this result seems reasonable.^30^ In contrast, the gesture imitation battery may have identified the MCI group as having more pronounced deficits in visuospatial cognitive function than in memory, with a higher likelihood of progressing to DLB or a posterior variant of ADD.^31^, ^32^ Unfortunately, we were unable to confirm that the MCI participants with low scores in this battery were in the prodromal stage of DLB or a posterior variant ADD.^33^ However, the addition of the gesture imitation battery to memory tasks may serve as a good screening tool for a wide range of patients with MCI.

In this study, we organised gesture imitation tasks for older adults and made several interesting observations. We investigated whether healthy older adults could accurately imitate gestures used in previous studies. In fact, 90% of the participants in the CN group successfully imitated most of the gestures. However, some gestures were found to be less successful in the CN group as well, with only a 63.4% success rate for G8. This rate was considerably lower than the 94.4% success rate for G8, reported by Tabuchi *et al*.^12^ This may be attributed to the differences in the demonstration methods between the two studies. In our study, the examiner only showed completed G8 gestures and did not show the step‐by‐step process. In the other study, Tabuchi *et al*.^12^ outlined the process of imitating a gesture in two steps, that is, step 1, shaping the same gesture in each hand, and step 2, twisting one hand and combining it with the other hand; no time limits were set for the task. Our findings suggest that when interpreting the performance of older adults in gesture imitation tasks, it is necessary to consider which gestures are used and how they are presented.

We examined the factors associated with performance in the gesture imitation and the Necker cube‐copying tasks and found that ageing and a diagnosis of dementia or MCI were commonly associated with lower scores in both tasks. However, sex and educational history were differentially implicated. In the gesture imitation task, male sex was associated with lower scores but not with educational attainment, whereas lower educational attainment was associated with lower scores but not with sex in the Necker cube‐copying task. Our results suggest that, although both the gesture imitation and Necker cube‐copying tasks are used to assess visuospatial/visuoconstructive functions, they have different task characteristics.

Educational history is known to be associated with cognitive performance,^34^, ^35^ but we found no significant association between the gesture imitation battery scores and years of education. In contrast, scores on the Necker cube‐copying task were significantly associated with years of education, similar to previous studies.^8^, ^21^ This discrepancy in results may be due to differences in how the two skills were acquired. The ability to copy the Necker cube is acquired through schooling,^21^ whereas imitating gestures, given their nature, may be acquired through play or other activities in daily life. Our results suggest that the gesture imitation battery may be more appropriate than the Necker cube‐copying task for identifying visuospatial/visuoconstructive dysfunction in older adults with insufficient educational attainment.

Male sex is usually associated with high performance in visuospatial/visuoconstructive tasks.^20^, ^36^ Interestingly, in the present study, high scores on the gesture imitation battery were significantly associated with the female sex. The exact mechanism by which females perform better in gesture imitation is unknown, but they may have a superior ability to imitate the actions of others. As gesture imitation is performed face‐to‐face, it requires observing the examiner's actions and imagining that one is performing them. The mirror neuron system (MNS) has been postulated to be a group of neurons that respond to the action of another individual as if the observer performed a similar action, and these are possibly associated with imitation behaviour.^37^ In normal subjects, suppression of the mu rhythm in electroencephalographs, a probable indicator of mirror neuron activity, was reported to be significantly weaker in males than in females when observing the hand movements of others.^38^ Sex‐based differences in mirror neuron activity may explain the sex‐based differences in gesture imitation performance, but the relationship between mu suppression and the MNS remains controversial.^39^ The relationship among gesture imitation performance, its neural substrates, and the MNS is an interesting neuropsychological topic that will be the focus of our future research.

This study has some limitations. First, people with ADD made up the majority of the dementia group, similar to another community‐based study.^40^ In contrast, only one patient had DLB, the second most common type of neurodegenerative dementia following ADD.^41^ People with ADD may be more likely to participate in epidemiological studies than people with other dementias, such as DLB and vascular dementia, because they have more preserved physical and motor functions. Given that visuospatial cognitive function is generally more strongly impaired in DLB than in ADD,^42^ caution should be exercised when directly applying the results of this epidemiological study to clinical practice. Second, the meaningful gestures used in this study are specific to Japan and may have no or different meanings in other countries; therefore, the results of this study cannot be directly applied to studies in other countries. Third, we did not estimate the test–retest or inter‐rater reliability of the gesture imitation battery. For this gesture imitation battery to be used in clinical practice, its reliability must be validated in future studies. The study used a battery of six gestures, but to develop a battery that can be administered in less time and is more useful for detecting MCI and dementia, future studies should also consider: (1) combinations that can be administered with fewer tasks; and (2) scoring methods that take into account patterns of error.

In conclusion, by combining gestures with set time limits, gesture imitation tasks showed high sensitivity and specificity for the detection of dementia. Gesture imitation tasks can be a low‐burden and highly effective method for detecting dementia, even in community medicine, such as during health check‐ups. However, gesture imitation tasks alone may not be sufficient to detect MCI; combining them with other cognitive tasks such as memory tasks may improve the detection rate of MCI.

## AUTHOR CONTRIBUTIONS

AT contributed to the study design, data collection, data analysis, and drafting and revision of the manuscript. MH, RF, SS, AK, and TI contributed to the study design, data collection, data analysis, and drafting and revision of the manuscript. MI contributed to the study design, data collection, and drafting and revision of the manuscript. SB and MT contributed to the study design and revision of the manuscript. All authors had final responsibility for the decision to submit the manuscript for publication. All authors read and approved the final version of the manuscript.

## ETHICS STATEMENT

The Research Ethics Committee of Kumamoto University approved this study (Kumamoto, Japan; approval number: GENOME‐333).

## PATIENT CONSENT

All participants provided written informed consent prior to data collection, in accordance with the Declaration of Helsinki.

## Supporting information


**Data S1.** Supporting Information.

## Data Availability

The data that support the findings of this study are available from the corresponding author upon reasonable request.
