# Supplementary material for: Gesture imitation performance in community‐dwelling older people: assessment of a gesture imitation task in the screening and diagnosis of mild cognitive impairment and dementia
Source: Psychogeriatrics. 2024 Jan 30;24(2):404–14. doi: 10.1111/psyg.13086 (PMC11577995; doi:10.1111/psyg.13086)
Supplement: Supplementary file 1 — Data S1. Supporting Information. [file PSYG-24-404-s001.docx]

**Appendix 1: Success rate of each gesture imitation and Necker cube-copying in the cognitively normal of each age**

|  | 65-74,  *n*=729 | 75-84,  *n*=401 | 85+,  *n*=54 | χ^2^ | *P*-value |
| --- | --- | --- | --- | --- | --- |
| Gesture imitation |  |  |  |  |  |
| G1 | 100.0% | 99.8% | 100.0% | ―^b^ | 0.384 |
| G2 | 97.8% | 97.3% | 96.3% | ―^b^ | 0.532 |
| G3 | 99.3% | 99.5% | 98.1% | ―^b^ | 0.409 |
| G4 | 98.5% | 98.0% | 98.1% | ―^b^ | 0.657 |
| G5 | 92.2% | 91.3% | 85.2% | ―^b^ | 0.199 |
| G6 | 89.7% | 85.5% | 75.9% | 11.4^a^ | *P* = 0.004^#, c, d^ |
| G7 | 91.6% | 86.5% | 90.7% | 7.4^a^ | 0.024 |
| G8 | 67.8% | 56.6% | 55.6% | 15.4^a^ | *P* < 0.001^#, c^ |
| Necker cube-copying | 83.7% | 71.8% | 59.3% | 34.1^a^ | *P* < 0.001^#, c, d^ |

^a^χ^2^ = The χ^2^ -test.

^b^Fisher's exact test.

^#^*P* < 0.017 (0.05/3) after Bonferroni correction.

^c^Significant difference between 65-74 and 75-84;

^d^Significant difference between 65-74 and 85+;

^e^Significant difference between 75-84 and 85+.

**Appendix 2: Success rate of each gesture imitation and Necker cube-copying in the cognitively normal of each sex**

|  | male,  *n*=438 | female,  *n*=746 | *P*-value^†^ |
| --- | --- | --- | --- |
| Gesture imitation |  |  |  |
| G1 | 99.8% | 100.0% | 0.370 |
| G2 | 98.2% | 97.2% | 0.335 |
| G3 | 99.5% | 99.2% | 0.718 |
| G4 | 98.2% | 98.4% | 0.817 |
| G5 | 91.8% | 91.4% | 0.914 |
| G6 | 84.7% | 89.4% | 0.022^#^ |
| G7 | 90.6% | 89.4% | 0.550 |
| G8 | 60.7% | 65.0% | 0.151 |
| Necker cube-copying | 81.1% | 77.1% | 0.123 |

^†^Fisher's exact test was used to determine the association between categorical variables.

^#^*P* < 0.05.

**Appendix 3: Success rate of each gesture imitation and Necker cube-copying in the cognitively normal of each educational level**

|  | ≤ 9 years,  *n*=282 | ≥ 10 years,  *n*=902 | *P*-value^†^ |
| --- | --- | --- | --- |
| Gesture imitation |  |  |  |
| G1 | 100.0% | 99.9% | 1.000 |
| G2 | 97.5% | 97.6% | 1.000 |
| G3 | 98.6% | 99.6% | 0.097 |
| G4 | 98.6% | 98.2% | 0.797 |
| G5 | 89.0% | 92.4% | 0.086 |
| G6 | 89.7% | 87.0% | 0.255 |
| G7 | 89.7% | 89.9% | 0.910 |
| G8 | 61.0% | 64.2% | 0.357 |
| Necker cube-copying | 66.0% | 82.5% | *P* < 0.001^#^ |

^†^Fisher's exact test was used to determine the association between categorical variables.

^#^*P* < 0.05.
